# Supplementary material for: Nanoparticle Delivery of Novel PDE4B Inhibitor for the Treatment of Alcoholic Liver Disease
Source: Pharmaceutics. 2022 Sep 7;14(9):1894. doi: 10.3390/pharmaceutics14091894 (PMC9501368; doi:10.3390/pharmaceutics14091894)
Supplement: Supplementary file 1 [file pharmaceutics-14-01894-s001.zip › pharmaceutics-1871591-supplementary.pdf]

Table S1. Primers for quantitative RT-PCR

Mouse CPT-1a	Forward	5’-GCTGCACTCCTGGAAGAAGA-3’
	Reverse	5’-GGAGGGGTCCACTTTGGTAT-3’
Mouse TNF-α	Forward	5’-GCCTCTTCTCATTCCTGCTTG-3’
	Reverse	5’-CTGATGAGAGGGAGGCCATT-3’
Mouse SOD1	Forward	5’-AAGCGGTGAACCAGTTGTG-3’
	Reverse	5’-TCCAACATGCCTCTCTTCATC-3’
Mouse SOD2	Forward	5’-GGCCAAGGGAGATGTTACAA-3’
	Reverse	5’-GCTTGATAGCCTCCAGCAAC-3’
Mouse IL-1β	Forward	5’-CTTTGAAGTTGACGGACCC-3’
	Reverse	5’-TGAGTGATACTGCCTGCCTG-3’
Mouse PPAR-α	Forward	5’-TCAGGGTACCACTACGGAGT-3’
	Reverse	5’-CTTGGCATTCTTCCAAAGCG-3’
Mouse PGC-1α	Forward	5’-ACAGCTTTCTGGGTGGATTG-3’
	Reverse	5’-CGCTAGCAAGTTTGCCTCAT-3’
Mouse ACOX1	Forward	5’-TCGAAGCCAGCGTTACGAG-3’
	Reverse	5’-ATCTCCGTCTGGGCGTAGG-3’
Mouse PDE4B	Forward	5'-AGCTCATGACCCAGATAAGTG-3'
	Reverse	5'-GCAGCGTGCAGGCTGTTGTGA-3'
Human Collagen I	Forward	5’-CCA TGC TGC CCT TTC TGC TCC TTT-3’
	Reverse	5’-CAC TTG GGT GTT TGA GCA TTG CCT-3’
Human α-SMA	Forward	5′-CCGACCGAATGCAGAAG GA-3′
	Reverse	5′-ACAGAGTATTTGCGCTCCGAA-3′
Human GAPDH	Forward	5′-TCG ACA GTC AGC CGC ATC TTC TTT-3′
	Reverse	5′-ACC AAA TCC GTT GAC TCC GAC CTT-3′
